# A machine learning model guided by physical principles for biofilter performance prediction

**DOI:** 10.1038/s41598-025-18585-8

**Published:** 2025-10-06

**Authors:** Uzma, Fabien Cholet, Dominic Quinn, Cindy J Smith, Siming You, William T Sloan

**Affiliations:** James Watt School of Engineering, University of Glasgow, Glasgow, G12 8QQ UK

**Keywords:** Organic carbon concentration, Biofilters, Physics-guided AI, Sparse dataset, Environmental biotechnology, Computational models, Machine learning

## Abstract

Despite the critical role of biofilters in water quality and sustainability, predicting their performance remains challenging due to the complexity of microbial interactions and limitations of sparse, high-dimensional datasets. Here, we introduce EnviroPiNet, a novel physics-guided AI framework designed to predict biofilter performance by accurately modeling carbon concentration dynamics. EnviroPiNet incorporates a physics-inspired backbone that enables the model to learn the physical properties of complex environments, ensuring predictions are grounded in system behavior. Additionally, we implement an ensemble hybrid approach to identify and extract key parameters essential for accurate carbon concentration predictions. We benchmark EnviroPiNet against conventional methods that lack physics-guided variable selection, demonstrating its superiority in identifying variables critical to biofilter performance evaluation. Trained on biofilter datasets, EnviroPiNet achieves a high coefficient of determination ($\text {R}^{2}$ = 0.9) on test sets, highlighting its predictive accuracy and robustness.

## Introduction

The success of artificial intelligence (AI) and machine learning (ML) methods depends heavily on computational power and the availability of diverse datasets^1^. With sufficient data and computational resources, AI can uncover patterns and anomalies, often imperceptible to traditional methods^2^. A long-standing goal in engineering is the creation of AI algorithms capable of accurately predicting the behaviour of unbuilt infrastructure or devices. This goal aligns with the foundational aim of engineering theory: to enable accurate prediction^3^. Before the advent of AI, predictive engineering relied on physics-based models, developed through ingenuity and experimentation^4^. These models have successfully informed the design of innovative structures and devices, from skyscrapers to spacecraft and energy-efficient household appliances^5^. However, environmental engineering has struggled to deliver predictive models with comparable reliability. For instance, when wastewater treatment technologies are implemented in new locations or applied to novel waste streams, theoretical models often fail to provide sufficient confidence for full-scale deployment. Instead, extensive pilot studies are conducted to optimize processes empirically before scaling up^6^. This disparity arises partly because environmental systems involve complex interactions between biological and environmental factors, which are difficult to model accurately. The biological processes at the heart of these systems are influenced by numerous environmental variables, many of which remain poorly understood^7^. Consequently, the development of reliable, physics-based predictive models for environmental technologies has been slow. Despite these challenges, advances in data collection methods, such as real-time monitoring and molecular microbiology, have made it possible to gather vast amounts of data on environmental systems^8^. These datasets often include high-resolution measurements of physical, chemical, and biological variables^9^. However, a lack of variability in experimental conditions often limits the diversity of these datasets, restricting their utility for fully data-driven models^10^. Sparse and non-exhaustive datasets make it challenging to develop predictive models that generalize well to unseen data^11^.

Dimensionality reduction techniques are commonly used to address these challenges by simplifying datasets while preserving essential information^12^. Methods like Principal Component Analysis (PCA) create orthogonal combinations of original variables, capturing most of the variance in a reduced set of components^13^. For more complex, nonlinear structures in data,Kernel Principal Component Analysis(kernel PCA) extends this approach by applying kernel functions to project the data into higher-dimensional feature spaces before performing PCA, enabling better separation of nonlinear relationships. Similarly, modern techniques like autoencoders use neural networks to encode nonlinear combinations of variables into a reduced feature set, ensuring that the essential characteristics of the data are retained^4^. These reduced features are then used to train predictive models, such as neural networks, with the aim of achieving robust performance on validation datasets. However, such methods remain entirely data-driven and may struggle with sparse or highly variable datasets. In the early 20th century, around the same time as PCA was first implemented, Engineers began to use a dimensional reduction technique, Buckingham Pi theory^14^, to make complex multivariate experimental data more tractable. Rather than being purely data driven this method relies on a modicum of understanding of the physics of the system and dimensional analysis, which merely dictates that the units of a formula must balance. If n variables in a system are measured and they are quantified using m dimensions (e.g. metres, seconds, kilograms,) then Buckingham Pi theory says that the system can be equally well described by n-m non-dimensional variables that are a combination of the originals. It turns out that this seemly innocuous finding is very powerful.

It transpired that the set of non-dimensional variables aids our understanding of the complex relationship between variables in physical systems. So, for example, in fluid dynamics the Reynolds number, Re, often appears as one the non-dimensional variables. Re indicates whether flows are laminar or turbulent and what the degree of turbulence is. Even for flow in channels that are orders of magnitude different in scale or shape, the same value of Re implies the same flow regime. Thus, Buckingham Pi theory is routinely used in designing scaled-down experiments of large-scale phenomena. In addition, for certain fluids and aeronautics problems if one assumes a mathematical model for a dependent variable that is the product of Buckingham Pi variables raised to an arbitrary power (monomial) it is possible to fit surprisingly effective models to experimental data. However, it has rarely been used in biological systems, and we have not seen it used as dimensional reduction method in combination with AI for modelling biotechnologies.

Buckingham Pi variables condense information in a reduced set of variables that, by dint of having no dimensions, are a function of properties related by the physics of the system. It could be argued that this is a more intuitive, and potentially, powerful way of reducing the dimensions of a data set than PCA or through the neural network bottlenecks used by autoencoders. In addition, by working with a reduced set of nondimensional variables it has been shown that models can make predictions using variable values that lie out with the range used in training^4^. This means that experimental results made at small-scale in the laboratory might be used to predict a similar system at a much larger scale used in a real application. In this study, we develop models to predict the concentration of organic carbon in the effluent from drinking water biofilters. We have elected to work with the sort of spare data that are common for biological experiments, where the range and combinations of engineering and biological conditions under which the filters were tested are a small subset of the possible conditions. The training data are from laboratory-scale biofilters, and the test data are from a full-scale operational biofilter. Our goal is to develop a predictive model for biofilter performance based on the physical properties of the variables to derive meaningful predictors for carbon concentrations. This approach is compared with state-of-the-art algorithms that lack physical property considerations, utilizing methods such as PCA, kernal PCA, and autoencoders to identify significant variables.

**Fig. 1:**
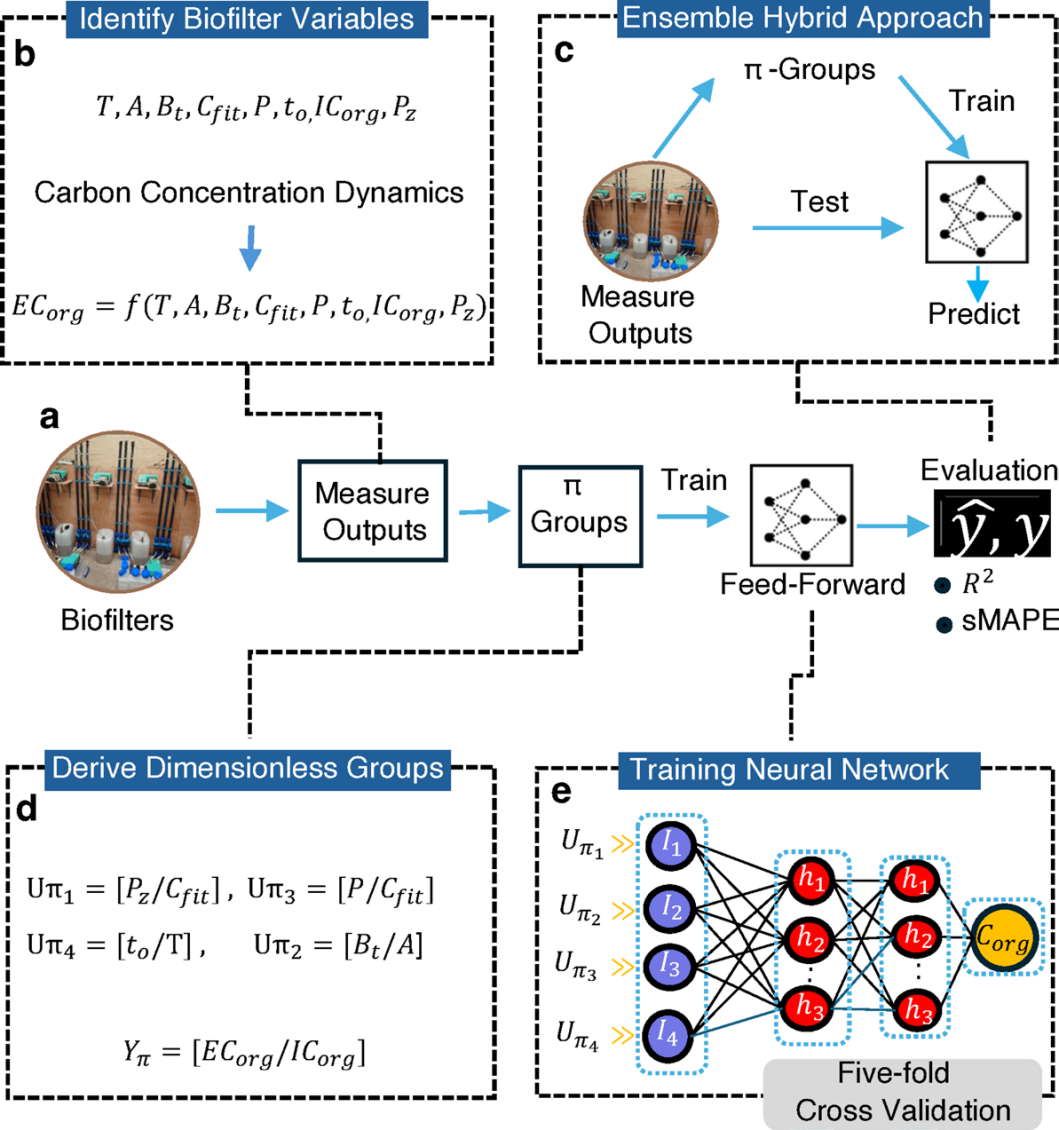
Overview of EnviroPiNet. (**a**) Schematic representation of the training process in EnviroPiNet. (**b**) Identification of biofilter variables and formulation of the carbon concentration function as a combination of independent variables. (**c**) Process of obtaining input from the biofilter, generating relevant variables, and feeding them into a neural network to predict carbon concentration. (** d**) Generation of dimensionless $\pi$-groups using the systematic approach of Buckingham Pi theorem. (**e**) Architecture of the feedforward neural network.

**Fig. 2:**
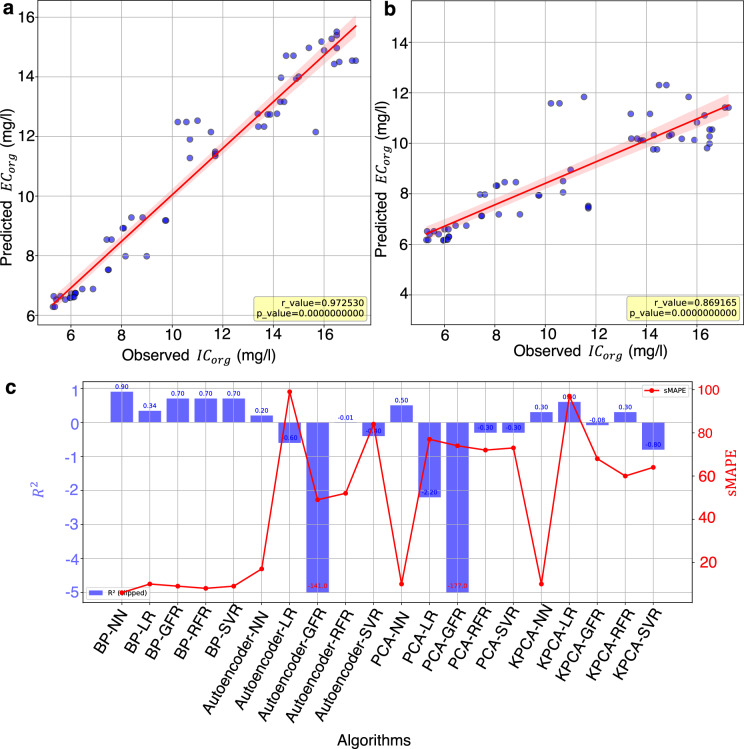
Regression plots and performance comparison between EnviroPiNet and BP_LR, highlighting predictive accuracy and model evaluation metrics. (**a**) Regression plot for EnviroPiNet with a Pearson correlation coefficient (r-value) of 0.97 (p < 0.05), indicating strong predictive accuracy. (**b**) Regression plot for BP_LR showing an r-value of 0.86 (p < 0.05), reflecting comparatively lower predictive accuracy. (** c**) Performance comparison on testing data using $\text {R}^{2}$ and symmetric mean absolute percentage error (sMAPE) metrics, with EnviroPiNet achieving higher $\text {R}^{2}$ and lower sMAPE, demonstrating superior performance over BP_GFR.

## Results

Figure 1a, provides a detailed visualization of the EnviroPiNet framework, showcasing how outputs from the biofilter are measured and processed.

The dataset was generated by collecting data from drinking water biofilters, capturing variables such as $T$, the temperature of the water at the sample location; $P_z$, the pore size of the sample, representing the characteristic diameter of the voids or pores within the biofilter material; $A$, the age of the filter measured as the time from the start of the experiment; $IC_{\text {org}}$, the influent carbon concentration of the sample; $EC_{\text {org}}$, the effluent carbon concentration of the sample; $B_t$, the empty bed contact time, which is estimated at the start of the experiment and assumed to be constant for each filter; $P$, the average diameter of the granular activated carbon (GAC) particles; $C_{\text {fit}}$, the diameter of the filter bed, which is a constant for each filter; $t_o$, the ambient air temperature, which is assumed to be constant for all locations in the bed at each time point. The units and dimensions of these parameters are shown in Table S1 (available in the Supplementary Methods). Each instance is a unique realisation of this vector of variables. These variables were systematically recorded over time to ensure robust and comprehensive data representation.

To evaluate the models’ predictions of organic carbon concentrations in the effluent, three time series datasets were combined for training. The time series are sparse; therefore, we combined all three datasets for training and set aside (20%) of the combined data, derived from the third dataset, for evaluation. The primary training dataset originates from Quinn , encompassing 175 observations over 162 days, with measurements of chemical composition, microbiology, and temperature taken weekly for the first 12 weeks and biweekly thereafter. Additionally, data from^15^ contributed 26 instances of the same observed variables. This study explored the impact of biofilter design and operational conditions on bacterial community composition and abundance in pilot-scale biofilters. Different filter media (granular activated carbon-sand versus anthracite-sand) and backwashing strategies were analyzed over 18 months of operation. The experimental setup included parallel operation of six replicate columns, ensuring robust assessments of the effects of media type and operational conditions. The dataset from^16^ added 116 samples, of which 54 observations were included in the training set. This study investigated five laboratory-scale biofilters with varying operational temperatures ($10^{\circ }$C and $20^{\circ }$C) over 48 weeks. The biofilters were packed with Norit® GAC 1240 and operated with an empty bed contact time of 3 h. Measurements of influent and effluent water quality parameters were taken weekly during the first 12 weeks and every six weeks thereafter. For testing, 62 observations from^16^ were reserved, forming a completely independent evaluation dataset. To ensure consistency, the dimensional data were transformed into dimensionless data using Equation S8. Details of the testing dataset are outlined in Details of the testing dataset are outlined in Table S2 (available in the Supplementary Methods).

In Fig. 1b, we highlight the identification and selection of key biofilter variables. The models relied on measured variables common to all studies, necessitating the exclusion of potentially useful information specific to individual studies. These variables were then used to formulate the equation, where the carbon concentration function is expressed as a combination of independent variables. In Fig. 1d, we illustrate the systematic approach of Buckingham Pi theorem to generate dimensionless $\pi$-groups from the selected variables. This approach simplifies the dataset while preserving the essential physical relationships between variables. The first part of EnviroPiNet generates $\pi$-groups (Equation S8), which are compared with two other methods, PCA (Eq. 2) and Autoencoder (Eq. 3), that reduce features to a smaller set of variables without preserving the physical relationships between them. The reduced variables are then used as inputs to a neural network or linear regression model, which determines the coefficients of the monomial model. Fig. 1c then highlights the process of feeding the $\pi$-groups into a neural network. After standardizations the $\pi$-groups, they are used as inputs to the neural network to predict carbon concentration, a crucial step for developing an accurate and generalizable predictive model. Finally, Fig. 1e, depicts the architecture of the feedforward neural network employed in EnviroPiNet. This architecture was designed to capture nonlinear relationships in the data, enhancing the model’s predictive performance for carbon concentration dynamics. The neural network training utilized 5-fold cross-validation, ensuring that after each epoch, a measure of goodness of fit was calculated for a randomly allocated 80% portion of the data used for training. Additionally, a second measure was computed using the remaining 20% of the data reserved for validation. These measures were plotted over the epochs to generate the loss plots. Supplementary Fig.S1 presents the loss plots for EnviroPiNet, using two metrics: Mean Squared Error (MSE) Supplementary Fig.S1.a and Mean Absolute Error (MAE) Supplementary Fig.S1.b. These curves illustrate the convergence of both training and validation measures, suggesting that the model effectively learns from the training data and generalizes well to unseen validation data. For the PCA-NN, KPCA-NN, and Autoencoder-NN models, the loss curves in supplementary Fig.S1.c and Fig.S1.e demonstrate that the models effectively learn from the training data and generalize well to the validation data. The corresponding Mean Absolute Error (MAE) values are presented in supplementary Fig.S1.d and Fig.S1.f, respectively.

However, as seen in Table 1, it does not perform well on unseen test data. This could be because, during training, the model is trained, tuned, or adjusted based on the validation performance. Since PCA and autoencoders focus on capturing the most significant variance in the data, they may ignore less prominent but still important features that are crucial for predicting the target variables. The proportion of variance captured by the first few principal components is shown in Fig.S2. Therefore, the validation data may exhibit the same general patterns as the training data, which allows the model to perform well during training. However, when we apply the model to completely unseen test data, the test data may contain patterns or variations that were not captured well by the dimensionality reduction techniques, leading to poor performance on the unseen data.The architecture and dimensionality reduction behavior of the autoencoder are illustrated in Fig.S3, which shows how eight input variables are compressed into four latent variables used for prediction. Typically, K-fold cross validations are not applied for regression analyses, rather a single goodness of fit is reported for the model using training data.

The trained models were applied to the unseen test dataset from study^16^. Thus, the reduced variates are calculated from the raw data and then the trained neural neworks, with trained node weights unchanged, and the monomial models, with the trained exponents, are applied. Again, we can calculate the various performance metrics can be calculated. The R2 and sMAPE metrics are compared for the models appled to the trainind and test datasets in Tables 1 and 2 respectively.

For Autoencoder reduced set of variates the relationship to the original variables can be nonlinear and is difficult to interpret in any biological or physical way. The reduced set of PCAs are orthogonal linear combinations of the orginal expanatory variables and thus are slightly easier to interpret in terms of the biology and physics. Buckingham Pi variates are, in effect, selected to encapsulate the science of the system. Thus, especially when used in an analytic functional form, their influence on the independent variable can be interpreted. The linear regression fitted exponents for the BP-LR are reported:

1 $$\begin{aligned} \frac{\textrm{EC}_{\textrm{org}}}{\textrm{IC}_{\textrm{org}}} = \text {intercept} \left( \frac{P_z}{C_{\textrm{fit}}} \right) ^{-0.06} \left( \frac{B_t}{A} \right) ^{-0.30} \left( \frac{P}{C_{\textrm{fit}}} \right) ^{0.53} \left( \frac{t_0}{T} \right) ^{-0.09} \end{aligned}$$

Where the intercept is the estimated constant, given by $\exp (-0.07) = 0.93$, and each term in the equation represents the effect of different variables on the organic carbon concentration in the treated water. The model suggests that changes in $\frac{P}{C_{\textrm{fit}}}$ have a large effect on the proportion of carbon removed in the filter. Thus, the smaller radius of GAC particles and the greater the radius of the filter (a surrogate for the amount of GAC) result in more carbon removed. Given that this variable relates to the surface area of activated carbon, this makes sense. The ratio $\frac{B_t}{A}$ is raised to the power $-0.3$, meaning that as $A$ increases, the proportion of carbon removed decreases, and thus, the older the filter, the poorer the treatment. Changes in $P_z$ and temperature have a smaller effect on carbon removal.

In addition to the results presented, Fig. 2a, b provide visual representations of the linear regression plots for the test dataset, comparing the EnviroPiNet and BP-LR models. Figure 2a showcases the Pearson correlation coefficient (r-value) of $0.97$ with $p < 0.05$ for EnviroPiNet, along with an R-squared value of 0.9. Figure 2b demonstrates an r-value of 0.86 with $p < 0.05$ for BP-LR, along with an R-squared value of 0.34. These figures emphasize the distinct characteristics and performance metrics of each model when evaluated on the test data.

In this study, we aimed to predict the carbon concentration $EC_{\text {org}}$,in drinking water biofilter effluent. The Environmental Buckingham Pi Neural Network (EnviroPiNet) demonstrated superior performance for predicting $EC_{\text {org}}$,achieving an $\text {R}^{2}$ value of 0.9, as shown in Table 1, and a sMAPE value of 6, as presented in Table 2. The model also yielded a Pearson correlation coefficient r -value) of 0.97 with a p-value less than 0.05, as depicted in Fig. 2a. The corresponding learning curve is illustrated in Fig. 1a. In comparison, linear regression (BP-LR) was applied, resulting in a Pearson correlation coefficient r-value of 0.86 with a p-value less than 0.05, as shown in Fig.2b, an $\text {R}^{2}$ value of 0.34, as presented in Table 1, and a sMAPE value of 10, as shown in Table 2, for the prediction of $EC_{\text {org}}$. Additionally, ensemble methods such as Gradient Forest Regression (BP-GFR), Random Forest Regression (BP-RFR), and Support Vector Regression (BP-SVR) were evaluated, each yielding a Pearson r r-value of approximately 0.7.

The results show that the EnviroPiNet model’s $\text {R}^{2}$ value outperformed the linear regression model. This suggests that the EnviroPiNet benefited from the Buckingham Pi theorem, enabling it to leverage the advantages of learning from a low-diversity dataset while using the neural network to capture nonlinear and complex relationships. Consequently, it outperformed linear regression in this specific application, as shown in Fig. 2c. An ablation study was conducted to assess the impact of physics-guided Buckingham Pi features on model performance. The EnviroPiNet neural network was trained on both the Buckingham Pi-transformed inputs and the original raw input variables. Performance metrics on the test set are summarized in Table S5. The model trained on Buckingham Pi groups achieved a higher $\text {R}^{2}$ and lower sMAPE compared to the model trained on raw variables, demonstrating the added value of physics-based feature engineering.To further examine the contribution of individual features, SHAP analysis was performed on the training data using a Ridge regression model with Buckingham Pi features. The SHAP summary and bar plots (Figs. S4 and S5) indicate that features $U\pi _2$ and $U\pi _4$ consistently contribute significant positive and negative impacts on predictions, respectively. Waterfall plots for individual observations (Fig.S6) reveal variation in feature contributions across samples, with $U\pi _2$ ranging from –0.19 to +0.38 and $U\pi _4$ from approximately –0.11 to +0.18. The alignment of SHAP values with the model’s predicted and expected outputs supports the physical relevance of these Buckingham Pi groups in predicting $\textrm{EC}_{\textrm{org}}$. Details about the SHAP analysis setup are provided in the Supplementary Methods.

**Table 1:** R-squared $(\text {R}^{2})$ values and standard deviations (in parentheses) used to evaluate the predictive performance of the models on both training and evaluation datasets, with results averaged across seven different seeds.

Comparison algorithm	Training	Testing
PCA-NN	0.9(0.01)	0.5(0.2)
PCA-LR	$-0.32$	$-2.22$
PCA-GFR	0.32	$-177$
PCA-RFR	0.9	$-0.3$
PCA-SVR	0.12	$-0.25$
KPCA-NN	0.75(0.03)	0.3(0.2)
KPCA-LR	$-0.5$	0.6
KPCA-GFR	0.6	$-0.08$
KPCA-RFR	0.8	0.3
KPCA-SVR	0.1	$-0.8$
Autoencoder-NN	0.8 (0.004)	0.2 (0.1)
Autoencoder-LR	$-0.21$	$-0.6$
Autoencoder-GFR	$-0.31$	$-141$
Autoencoder-RFR	0.9	$-0.01$
Autoencoder-SVR	$-0.1$	$-0.4$
BP-NN	0.95 (0.005)	0.9 (0.01)
BP-LR	0.4	0.34
BP-GFR	0.9	0.7
BP-RFR	0.9	0.7
BP-SVR	0.6	0.7

EnviroPiNet is compared with other state-of-the-art algorithms using Python-based implementations. The values indicate model performance on training and testing datasets, highlighting predictive accuracy and consistency

**Table 2:** sMAPE and standard deviation (in parentheses) for the neural network model trained on the dataset, evaluated on both training and evaluation datasets, with results averaged across seven different seeds.

Comparison algorithm	Training	Testing
PCA-NN	10(0.7)	10(6.3)
PCA-LR	77	77
PCA-GFR	42	42
PCA-RFR	72	72
PCA-SVR	45	73
KPCA-NN	18 (1.1)	10 (5)
KPCA-LR	97	97
KPCA-GFR	68	68
KPCA-RFR	60	60
KPCA-SVR	64	64
Autoencoder-NN	12 (1)	17 (5)
Autoencoder-LR	94	99
Autoencoder-GFR	38	49
Autoencoder-RFR	19	52
Autoencoder-SVR	84	84
BP-NN	2 (0.1)	6 (2.1)
BP-LR	18	10
BP-GFR	0.9	0.7
BP-RFR	0.9	0.7
BP-SVR	0.6	0.7

The comparison highlights the performance of EnviroPiNet against other state-of-the-art algorithms using Python-based implementations. The sMAPE values and standard deviations reflect the accuracy and reliability of the models on training and testing datasets

## Discussion

In discussing our findings, it’s essential to underscore the significance of our comparative analysis across various dimensionality reduction techniques and modeling approaches. By systematically evaluating methods such as BP, PCA, kernal PCA and autoencoder, in conjunction with neural networks or a monomial function of the reduced variates, we gain valuable insights into their respective strengths and weaknesses in predicting carbon concentrations in drinking water biofilter effluent.

PCA,kernal PCA, and autoencoder methods are generally designed to reduce dimensionality without necessarily considering the physical or interpretive meaning of the features. As a result, they might produce feature combinations that aren’t as relevant for predicting carbon concentrations in water, leading to suboptimal generalization. However, the Buckingham Pi approach maintains physical relevance, which helps the model understand and learn the meaningful relationships in the data. This results in better generalization and performance on test data.Our assessment comprised two distinct analyses aimed at scrutinizing the efficacy of the model. Employing R-squared ($R^2$) as a performance metric (Table 1), we observed promising outcomes when employing the Buckingham Pi theorem for dimensionality reduction followed by neural network application. Specifically, EnviroPiNet achieved an $R^2$ value of 0.9, outperforming PCA (0.5), KPCA 0.3 and autoencoder (0.2). Even when linear regression was applied post-reduction, BP still demonstrated favorable performance across all regression methods (Table 1), with an value of 0.7 compared to PCA (-177), KPCA (-0.08) and Autoencoder (-141) under GFR. These findings underscore the superior dimensionality reduction capabilities of BP in predicting carbon concentrations in effluent water, especially when combined with neural network techniques .Analysis using sMAPE as a performance metric (Table 2) demonstrated that Buckingham Pi, followed by ANN (EnviroPiNet), exhibited the best performance, achieving an sMAPE value of 6. Comparatively, PCA, KPCA and autoencoder achieved higher sMAPE values of 6, 10 and 17, respectively. A detailed explanation of the performance difference between Autoencoder-NN and Autoencoder-LR is provided in the Supplementary Results. Similarly, Buckingham Pi followed by linear regression (BP-GFR) also performed better, achieving an sMAPE value of (8), outperforming PCA (74), KPCA (68), and autoencoder (49).

Furthermore, our comparative analysis highlights that while $R^2$ reflects the overall predictive accuracy of the models, sMAPE provides insights into the accuracy of individual predictions. In both performance metrics, Buckingham Pi (BP) consistently outperformed other methods, demonstrating its strengths in predictive accuracy and individual prediction precision.

The study^17^ supports the preference for $R^2$ over sMAPE when comparing predicted and measured values of continuous variables. $R^2$ offers bounded values that are interpretable and comparable across datasets, making it a more consistent metric for evaluating the overall performance of predictive models, particularly when the focus is on general trends and fit rather than individual prediction accuracy.

Implications extend beyond academia to real-world applications in water quality management. Accurate prediction of organic carbon concentrations serves as an early warning system for potential contaminants in drinking water, enabling proactive measures to safeguard public health and optimize treatment processes. Importantly, while these dimensionality reduction techniques effectively predict dimensionless variables (Pi variables), post-processing is necessary to revert the predictions into meaningful dimensional variables such as $EC_{\text {org}}$. This step highlights the practical implications of these predictions, which indicate that EnviroPiNet showed a higher correlation and a better $R^2$ value of 0.9, indicating that the neural network was able to capture the relationship between the predictors and the target variable more effectively than BP-LR in this case.

Our study has provided va luable insights, but future research should focus on evaluating the model across different test datasets to assess its robustness and generalizability. This will ensure comprehensive evaluations and enhance the applicability of our approach. Since this is the first time the model has been applied in biofilters, future work could explore its application to similar systems or processes to further validate its predictive capabilities and expand its scope. Additionally, refining the model architecture and incorporating advanced techniques could improve its predictive performance.

By addressing these limitations and pursuing avenues for future research, we can continue to advance our understanding of water quality management and contribute to the development of more effective and sustainable treatment strategies.

## Methods

## Biofilter system description and dataset collection

This study utilized data from three independent drinking water biofilter studies that varied in design configuration, operational conditions, and sampling duration. Across all systems, influent and effluent organic carbon concentrations were monitored over time, along with key media and environmental characteristics such as pore size, GAC particle diameter, filter age, and ambient temperature. To ensure consistency across studies, we retained only variables common to all three datasets: water temperature, influent and effluent organic carbon concentrations, filter age, pore size, granular media characteristics, ambient temperature, and contact time. A complete list of variables, including units and dimensions, is provided in the Supplementary Methods.

A combined dataset of 317 observations was compiled, of which 20% (62 samples) all sourced from the third study was reserved for model testing. The training dataset included 175 samples from a laboratory-scale study by Quinn^18^ conducted over 162 days, 26 samples from a pilot-scale study^15^ focused on media and backwashing effects on microbial communities, and 54 samples (out of 116 total) from a temperature-gradient study^16^ involving five laboratory-scale biofilters. All filters used granular activated carbon (GAC) media with varying configurations and operational strategies. Detailed descriptions of the biofilter systems, data sources, experimental setup, and sampling procedures are provided in the Supplementary Methods to ensure reproducibility.

## Data integration and preprocessing

The three biofilter datasets were combined by selecting only variables common to all studies, ensuring consistency of measured features. Because the original datasets had varying sampling intervals and experimental durations, no direct temporal alignment of timestamps was performed. Instead, each data point was treated as an independent observation representing a unique combination of biofilter conditions and time. Time-dependent patterns were not explicitly modelled; the focus was on predicting effluent organic carbon concentrations from concurrent input variables. Missing data were handled by excluding samples with incomplete measurements for key predictor or response variables to avoid bias introduced by imputation. This resulted in a clean, harmonized dataset used for training and testing. To ensure the generalizability of our models across different biofilter scales, we employed dimensional analysis using the Buckingham Pi theorem to transform the original input variables into dimensionless groups. This approach normalizes the data relative to biofilter size and operational conditions, thereby reducing scale-related biases. Further methodological details are provided in the Supplementary Methods section titled *Dimensionless Variable-Based Modelling of Biofilter Performance*. Model training and validation were conducted on these dimensionless variables, enabling robust, cross-scale predictions. After model inference, the predicted dimensionless outputs were converted back into their original dimensional form such as effluent organic carbon concentration $EC_{\text {org}}$ to allow for practical interpretation. This procedure ensures that model performance metrics reflect true predictive capability across systems of varying scale.

## Modelling with PCA, kernal PCA, and autoencoder dimensions

To reduce the dimensionality of the original 8 independent variables, we employed both the Buckingham Pi dimensional analysis and advanced data-driven methods: PCA, kernal PCA, and Autoencoder-based feature extraction. The Buckingham Pi method generated 4 dimensionless variables from the original set. This Complementing this, PCA was applied to the same dataset, retaining the first four principal components, $\{\text {PCA}_1, \dots , \text {PCA}_4\}$ as reduced variates. To capture potential nonlinear structures in the data, kernel PCA was also implemented using a radial basis function (RBF) kernel. The first four kernel principal components $\{\text {KPCA}_1, \dots , \text {KPCA}_4\}$ were selected to represent the transformed feature space. Similarly, an Autoencoder was designed with a bottleneck layer comprising 4 neuron $\{h_1, \dots , h_4\}$, which served as nonlinear latent features representing a compressed version of the original inputs. For all three types of reduced variebles( PCA, kernal PCA, and Autoencoder), neural network models were constructed to approximate arbitrary mapping functions $p$ and $r$, $q$ such that:

2 $$\begin{aligned} \begin{aligned} \text {EC}_{\text {org}}&= p(\text {PCA}_1, \text {PCA}_2, \text {PCA}_3, \text {PCA}_4), \\ \text {EC}_{\text {org}}&= r(\text {KPCA}_1, \text {KPCA}_2, \text {KPCA}_3, \text {KPCA}_4) \end{aligned} \end{aligned}$$

and

3 $$\begin{aligned} \text {EC}_{\text {org}} = q(h_1, h_2, h_3, h_4). \end{aligned}$$

In all three cases for $p$ $r$ and $q$, we used a neural network constructed in exactly the same way as for the Buckingham Pi analysis. These models are referred to as PC-NN,KPC-NN and Autoencoder-NN. For completeness, we also use a monomial model for $p$ and $q$ in equations (2) and (3), which we refer to as PC-LR, KPC-LR and Autoencoder-LR, respectively. We additionally experimented with ensemble-based regressors including Random Forest Regression (RFR), Gradient Boosted Regression (GRF), and Support Vector Regression (SVR). A full account of these models, including performance comparisons and hyperparameter configurations, is provided in the **Supplementary Methods**.Unlike PCA, which provides orthogonal linear combinations of input variables, the autoencoder generates nonlinear latent features that are more difficult to interpret in a physical or biological context (see Fig.S3 for architecture and input mappings).

## Regression models for predictive analysis

Following dimensionality reduction, four regression models were applied to evaluate the predictive performance of the compressed feature sets: Ridge Regression, Support Vector Regression (SVR), Random Forest Regression (RFR), and Gradient Boosting Regression (GBR). These models were selected for their complementary strengths in handling linear and nonlinear relationships, robustness to overfitting, and ability to capture complex patterns. All models were implemented using the scikit-learn library and trained on the reduced training data.

### Ridge regression

Ridge Regression^19^ is a linear model that employs L2 regularization to mitigate overfitting and address multicollinearity. It minimizes the residual sum of squares while penalizing large coefficient values, resulting in a more stable model with improved generalization. In our study, ridge regression was applied to the reduced feature sets obtained through dimensionality reduction. Hyperparameter tuning was conducted on the regularization strength parameter $\alpha$, with candidate values logarithmically spaced between $10^{-3}$ and $10^{10}$. A five-fold cross-validation scheme was employed to identify the optimal $\alpha$ by minimizing the mean squared error (MSE) on the validation sets. To maintain consistency with the preprocessing pipeline and ensure standardized feature scaling, the model was configured with fit_intercept=False.

### Gradient boosting regression

Gradient Boosting Regression^20,21^ is an ensemble learning method that constructs a strong predictive model by sequentially combining multiple weak learners, typically decision trees. At each iteration, a new model is trained to correct the residual errors of the previous model by minimizing a differentiable loss function using gradient descent. This iterative optimization enables the model to capture complex nonlinear relationships and subtle patterns in the data. In this study, Gradient Boosting Regression was implemented using the scikit-learn library. The model was configured with 100 estimators, a learning rate of 0.1, and a fixed random seed (42) to ensure reproducibility. These hyperparameters were selected based on standard best practices and preliminary experimentation to balance model complexity and generalization performance.

### Random forest regression

Random Forest Regression^22^ machine is an ensemble learning technique that builds a collection of decision trees and averages their predictions to enhance generalization and mitigate overfitting. Each tree is trained on a bootstrap sample of the data, and at each node, a random subset of features is considered for splitting. This strategy reduces correlation between trees and improves model robustness, enabling the algorithm to effectively capture complex nonlinear relationships with minimal feature engineering. In this study, the Random Forest Regressor was implemented using the scikit-learn library with 100 estimators and a fixed random seed (42) to ensure reproducibility.

### Support vector regression (SVR)

Support Vector Regression (SVR)^23^ extends the foundational principles of Support Vector Machines (SVM) to continuous output prediction tasks. It aims to identify a function that approximates the true target values within a predefined margin of tolerance $\varepsilon$, while simultaneously minimizing model complexity. This is accomplished by fitting a hyperplane that optimally balances accuracy and generalization, effectively controlling the trade-off between prediction precision and overfitting.Support Vector Regression (SVR) is particularly effective at capturing nonlinear relationships, especially when used in conjunction with kernel functions. In this study, the Radial Basis Function (RBF) kernel was employed to enable the model to learn complex, nonlinear patterns in the dataset. The SVR model was implemented using the scikit-learn library with the following configuration: kernel=’rbf’, regularization parameter $C = 1.0$, and $\varepsilon = 0.1$. All models were trained on the dimensionally reduced training data and evaluated using independent test sets. Model performance was assessed using the coefficient of determination ($R^2$) and the symmetric mean absolute percentage error (sMAPE), enabling a robust comparison across different regression techniques. Hyperparameter tuning for each model was performed using cross-validation to optimize predictive accuracy and prevent overfitting.

## Model development

Once the training and testing datasets were prepared, we applied four different dimensionality reduction techniques Buckingham Pi (BP) theorem, PCA, Kernel PCA (KPCA), and Autoencoder-based reduction to reduce the feature space and enhance model interpretability and performance. These methods were then integrated with neural networks and conventional regression models to evaluate predictive accuracy across a range of modelling paradigms. The resulting hybrid models include neural network-based variants EnviroPiNet (BP-NN), PCA-NN, KPCA-NN, and Autoencoder-NN and combinations with regression models such as Linear Regression (LR), Gaussian Forest Regression (GFR), Random Forest Regression (RFR), and Support Vector Regression (SVR). For example, the PCA-transformed dataset was used to train four distinct models: PCA-LR, PCA-GFR, PCA-RFR, and PCA-SVR. This strategy enabled us to systematically compare the effectiveness of dimensionality reduction techniques when coupled with both machine learning and statistical modelling approaches.

## Neural network architecture and training

A purely data-driven approach adopted in this study involves the use of a feedforward neural network. Using the same reduced, non-dimensional training dataset, the input variables were first standardized to have zero mean and unit variance. The scaled independent variables were used as inputs to a three-layer feedforward neural network, as outlined in the papers^24,25^. The architecture consists of one input layer, two hidden layers, and one output layer. Rectified Linear Unit (ReLU) activation functions were used in the hidden layers, while a linear activation function was employed in the output layer to accommodate continuous-valued predictions. Details of the architecture and hyperparameters are provided in Supplementary Table S4. For consistency and reproducibility, the number of random seeds was set to 7, which controls random processes such as weight initialization and data shuffling. Model training was carried out using the Adaptive Moment Estimation (Adam) optimizer, known for its adaptive learning rate and efficiency in training deep networks. To evaluate the model’s generalization capability and reduce the risk of overfitting, we implemented a 5-fold cross-validation strategy. In this process, the training dataset was divided into five subsets; each fold served once as a validation set while the remaining four were used for training. This validation procedure was repeated across all folds, with model fitting performed in each epoch. To further enhance generalization, an L2 kernel regularization term was applied during training. This penalizes large weights and encourages smoother model behaviour refer to the application of neural networks to Buckingham Pi-reduced inputs as BP-NN (Buckingham Pi-Neural Network).

## Measures of performance

The proposed model’s evaluation employs both the Symmetric Mean Absolute Percentage Error (sMAPE) and $R^2$ metrics^17^ to provide comprehensive insights into its performance. By considering multiple evaluation metrics, we can better assess the model’s accuracy and generalization capability across different datasets and scenarios.

## R-squared ($R^2$)

During training, the model learns the relationships between the input variables and the target variable by adjusting its parameters to minimize the prediction error, typically measured using the Mean Squared Error (MSE). The $R^2$ metric is utilized to evaluate the performance of the designed predictive model. The formula for $R^2$ is given by:

4 $$\begin{aligned} R^2 = 1 - \frac{\sum _{i=1}^n (y_i - \hat{y}_i)^2}{\sum _{i=1}^n (y_i - \bar{y})^2}, \end{aligned}$$

where $y_i$ are the observed values, $\hat{y}_i$ are the predicted values, and $\bar{y}$ is the mean of the observations. $R^2$, also known as the coefficient of determination, quantifies the proportion of the variance in the dependent variable that is predictable from the independent variables. Its value ranges from 0 to 1, with a value of 1 indicating a perfect fit, where the model explains all the variability in the dependent variable. A value of 0 suggests that the model does not explain any of the variability, while negative values indicate that the model fits the data worse than a horizontal line. $R^2$’s bounded nature facilitates consistent interpretation across different datasets and applications, making it particularly valuable when comparing predicted and measured values of continuous variables.

## Symmetric mean absolute percentage error (sMAPE)

In addition to $R^2$, the Symmetric Mean Absolute Percentage Error (sMAPE) is employed as a metric to evaluate the model’s performance. sMAPE offers advantages such as simplicity and computational efficiency. It is calculated as:

5 $$\begin{aligned} \text {sMAPE} = \frac{100}{n} \sum _{i=1}^n \frac{|y_i - Y_i|}{|y_i| + |Y_i|}, \end{aligned}$$

where $y_t$ represents the true value and $Y_t$ represents the predicted value, and $n$ is the number of samples. However, sMAPE values may vary significantly from one dataset to another, making direct comparisons challenging.

## Supplementary Information

Supplementary Information.

## Data Availability

The raw dataset’s accession number is currently unavailable, as it has not yet been published; however, all relevant details are provided in Quinn’s thesis^18^. Information from the dataset referenced in study^15^ has also been incorporated in this work. Additionally, the dataset described by Shi et al. ^16^ has been used. These datasets have been analysed as part of this study, and the resulting data, along with the EnviroPiNet-generated dataset and the ensemble results of BP-LR, have been uploaded to the CodeOcean repository. All analyzed datasets can be accessed at https://codeocean.com/capsule/5400466/tree with Doi is https://doi.org/10.24433/CO.7617036.v1.
