# Supplementary material for: A machine learning model guided by physical principles for biofilter performance prediction
Source: Sci Rep. 2025 Oct 6;15:34664. doi: 10.1038/s41598-025-18585-8 (PMC12500875; doi:10.1038/s41598-025-18585-8)
Supplement: Supplementary file 1 — Supplementary Information. [file 41598_2025_18585_MOESM1_ESM.pdf]

Supplementary Material

Uzma[1*], Fabien Cholet[1], Domenic Quinn[1], Cindy Smith[1], Siming You[1] and William Sloan[1]

[1] James Watt School of Engineering, University of Glasgow, Glasgow, G12 8QQ, UK

Corresponding author email: uzma.k.khan@glasgow.ac.uk

Supplementary Methods

Biofilter System Description and Data Collection

This study uses data collected from drinking water biofilters that vary in scale and operational conditions. Key system characteristics including filter dimensions, temperature, media properties, filter age, pore size, GAC particle diameter, ambient air temperature, empty bed contact time, and influent and effluent water quality were recorded. The dataset includes measurements of effluent organic carbon concentrations along with associated predictor variables, collected over multiple time points.

To evaluate the model predictions of effluent organic carbon concentrations, three experimental time series datasets from drinking water biofilters were used. These datasets capture biofilter operation under various design configurations, environmental conditions, and time scales. Because the time series were relatively sparse, we combined all three datasets to enrich the training data and withheld 20% of the combined dataset taken from the third dataset for model testing. The input features were harmonized across studies, so only variables common to all three datasets were retained. The description of each dataset is provided below.

Training Dataset Description

Quinn [1] monitored the chemical composition, microbiology, and temperature in the influent effluent and at various depths in a suite of laboratory biofilters. The biofilters were operated for a period of 162 days. Measurements were taken weekly for the first 12 weeks, followed by biweekly measurements thereafter. The filter material was granular activated carbon with almost uniformly dimensioned spherical particles. Ultimately the dataset comprised 175 observations of a vector of variables: T, the temperature of the water at the sample location; Pz, the pore size of the sample, representing the porosity or void space in the biofilter material; A, the age of the filter measured as the time from the start of the experiment; IC_{org}, the influent

carbon concentration of the sample; EC_{org}, the effluent carbon concentration of the sample; B_t, the empty bed contact time, which is estimated at the start of the experiment and assumed to be constant for each filter; P, is the average diameter of the GAC particles; C_{fit}, is the diameter of the filter bed, which is a constant for each filter; t_o , is the ambient air temperature, which is assumed to a constant for all locations in the bed at each time point. The units and dimensions of these parameters are shown in Table S1. Each instance is a unique realisation of this vector of variables.

In addition to the Quinn [1] dataset, data from the [2] were incorporated, comprising a total of 26 instances of the same observed variables. The aim of the study was to investigate the effects of biofilter design and operating conditions on the bacterial communities within the filter media and their relationship to the microbiome in the filter effluent water. Specifically, the research focused on how different filter media (granular activated carbon (GAC)-sand versus anthracite-sand) and backwashing strategies (chloraminated versus nonchloraminated water) influenced the bacterial community composition and abundance in pilot-scale biofilters.

The study was conducted using pilot-scale biofilter columns, simulating real filter conditions in a controlled laboratory environment. The experimental setup included six replicate columns operated in parallel over an extended period of 18 months. This parallel operation and the reproducibility of sequencing results across the replicate columns enabled a robust assessment of the differences in bacterial communities attributable to the filter media type and operational conditions.

The study from [3], were. also incorporated, comprising 116 samples, of which 54 observations were used for training. This study was conducted on

five laboratory-scale biofilters (A, B, C, D and E) packed with GAC (Norit® GAC 1240) which were operated for 48 weeks at 10°C and 20°C degrees in temperature-controlled rooms. Each biofilter had a bed length of 30cm and an internal diameter of 2.6cm and were operated with an EBCT of 3H. All biofilters were fed with the same influent water sourced from a local freshwater reservoir (Pateshill Water Treatment Works, Scottish Water, United Kingdom). Immediately upon collection, the water was filtered through 10 μm polypropylene cartridge filters (Spectrum, UK) and stored in acid-washed and 18.2 M Ohm deionised water rinsed jerry cans at 4◦C until its use for the first 12 weeks, water quality parameter was measured weekly in influent and effluent water. For the remaining 36 weeks, influent and effluent water quality parameters were measured every 6 weeks.

The training dataset combines data from [1,2,3] to enhance its richness and diversity, thereby enabling more robust predictive modelling.

Testing Dataset

To test the models, 20 % of the combined data, derived from the study [3] was set aside. This dataset comprises 62 observations of a vector of variables, which are completely independent of the training dataset. The detail of the test dataset is mentioned in Table S2. The dimensionless target training data is formed by substituting the dimensional training data into equation (S8). The same process is repeated for the dimensionless test data.

Table S1: Units and Dimensions of Variables Used in the Training Data.

Variable	Range	Units	Dimensions
Temperature (T)	[4, 28]	k	$L^0.M^0.T^0.K^1$
Pore size (Pz)	[0.1,0.2]	nm	$L^1.M^0.T^0.K^0$
Age (A)	[7, 539]	days	$L^0.M^0.T^1.K^0$
Influent Org − CCon (IC_{org})	[3.824, 17]	mg/L	$L^{-3}.M^1.T^0.K^0$
Empty Bed Contact time (B_t)	[8.4,450]	Sec	$L^0.M^0.T^1.K^0$
GAC particle diameter (P)	[0.9,1.058]	mm	$L^1.M^0.T^0.K^0$
Filter diameter (C_{fit})	[3.75,2.6]	mm	$L^1.M^0.T^0.K^0$
Ambient Temperature (t_o)	37	k	$L^0.M^0.T^0.K^1$
ECorg (EC_{org})	[0.4,12.729]	mg/L	$L^{-3}.M^1.T^0.K^0$

Note: Variables represent key parameters in the training dataset, with their ranges, units, and dimensions provided.

Table S2: Units and Dimensions of Variables Used in the Testing Data.

Variable	Range	Units	Dimensions
Temperature (T)	[10, 20]	k	$L^0.M^0.T^0.K^1$
Pore size (Pz)	0.1	nm	$L^1.M^0.T^0.K^0$
Age (A)	[70, 340]	days	$L^0.M^0.T^1.K^0$
Influent Org − CCon (IC_{org})	[7.725, 21.7]	mg/L	$L^{-3}.M^1.T^0.K^0$
Empty Bed Contact time (B_t)	180	Sec	$L^0.M^0.T^1.K^0$
GAC particle diameter (P)	1.058	mm	$L^1.M^0.T^0.K^0$

Filter diameter (C_{fit})	2.6	mm	$L^1.M^0.T^0.K^0$
Ambient Temperature (t_o)	37	k	$L^0.M^0.T^0.K^1$
ECorg (EC_{org})	[5.291,17.25]	mg/L	$L^{-3}.M^1.T^0.K^0$

Note: Variables represent key parameters in the testing dataset, with their ranges, units, and dimensions provided.

Dimensionless variable-Based modelling of biofilter performance

Following the procedure detailed in the study [4], we implemented the Buckingham Pi theorem.

Although Buckingham Pi dimension reduction is well-documented, it is not commonly applied in biotechnologies or bioinformatics and involves subjective choices. Furthermore, in engineering contexts, the resulting dimensionless variables are often incorporated into predictive models with predefined functional forms, such as monomials, rather than AI models. The process is outlined as follows:

Initialization: We postulate that an unknown function, f , relates the dependent variable of interest, organic carbon concentration C_{org} , to a vector of independent physical variables, x =($x_1, x_2, \dots . x_n$):

$$EC_{org} = f(T, A, B_t, C_{fit}, \mathrm{P}, t_o, IC_{org}, P_z) \qquad \text{(S1)}$$

Define base subset: If the data are described by m dimensions, then select a subset of m independent variables whose units span all m dimensions. In this case there are 4 fundamental dimensions, L, T, M, and K and a base subset of 4 variables with unique units that encapsulate all the dimensions are T, IC_{org}, A and C_{fit} (Table 1).

If the data are described by m dimensions, then select a subset of m independent variables whose units span all n dimensions. In this case, there are 4 fundamental dimensions: L, M, T and K . A base subset of 4 variables with unique units that encapsulate all the dimensions is T IC_{org}, A and C_{fit} (Table S1).

$$\text{Base subset} = \{\, T, IC_{org}, A, C_{fitt} \} \tag{S2}$$

Dimensional Analysis: For the remaining m independent variables, assume that their dimensions can be expresses as products of powers of the base subset dimensions. In our case, the remaining 5 variables,

$$[P_z] = [T^a IC_{org}{}^b A^c\, C_{fit}{}^d] \;,$$
$$[B_t] = [T^e IC_{org}{}^f A^g\, C_{fit}{}^h] \;, \tag{S3}$$
$$[\mathrm{P}] = [T^i IC_{org}{}^h A^k\, C_{fit}{}^l] \;,$$
$$[t_o] = [T^m IC_{org}{}^n A^o\, C_{fit}{}^p] \;,$$
$$[\mathrm{E}Corg] = [T^q IC_{org}{}^r A^s\, C_{fit}{}^t] \,,$$

where, $\{a,b,....,t\}$ are arbitrary, as yet, unknown real values. Given equation (S4), we can define a new set of variables as follows:

$$U\pi_1 = \frac{P_z}{T^a\; IC_{org}{}^b A^c\, C_{fit}{}^d} \,,$$
$$U\pi_2 = \frac{B_t}{T^e IC_{org}{}^f A^g\, C_{fit}{}^h} \,,$$
$$U\pi_3 = \frac{\mathrm{P}}{T^i IC_{org}{}^j A^k\, C_{fit}{}^l} \,, \tag{S4}$$
$$U\pi_4 = \frac{t_o}{T^m IC_{org}{}^n A^o\, C_{fit}{}^p} \,,$$
$$Y_\pi = \frac{ECorg}{T^q IC_{org}{}^r A^s\, C_{fit}{}^t} \;.$$

By insisting the dimensional equivalence in equations (S3) we then need to select exponents $\{a,b,....,t\}$ that ensure the new variables are dimensionless.

This becomes an exercise in linear algebra. So, we require

$$[\mathrm{U\pi_1}] = \frac{\mathrm{L}^1}{(\mathrm{K})^{\mathrm{a}}.(\mathrm{L}^{-3}.\mathrm{M}^1)^{\mathrm{b}}.(\mathrm{T})^{\mathrm{c}}.\mathrm{L}^{\mathrm{d}}} = \left(\mathrm{L}^{1+3\mathrm{b}-\mathrm{d}}.\,\mathrm{M}^{-\mathrm{b}}.\,\mathrm{K}^{-\mathrm{a}}.\,\mathrm{T}^{-\mathrm{c}}\right) = 1$$

$$[\mathrm{U\pi_2}] = \frac{\mathrm{T}^1}{(\mathrm{K})^{\mathrm{e}}.(\mathrm{L}^{-3}.\mathrm{M}^1)^{\mathrm{f}}.(\mathrm{T})^{\mathrm{g}}.\mathrm{L}^{\mathrm{h}}} = \left(\mathrm{L}^{3\mathrm{f}-\mathrm{h}}.\,\mathrm{M}^{-\mathrm{f}}.\,\mathrm{K}^{-\mathrm{e}}.\,\mathrm{T}^{1-\mathrm{g}}\right) = 1$$

$$[U\pi_3] = \frac{L^1}{(K)^i.(L^{-3}.M^1)^j.(T)^k.L^l} = \left(L^{1-3j-1}.M^{-j}.K^{-i}.T^{-k}\right) = 1 \quad \text{(S5)}$$

$$[U\pi_4] = \frac{(K^1)}{(K)^m (L^{-3}.M^1)^n (T)^o (L)^p]} = (L^{3n-p}.M^{-n}.K^{1-m}.T^{-o}) = 1$$

$$[Y_\pi] = \frac{L^{-3}.M^1}{(K)^q.(L^{-3}.M^1)^r.(T)^s.L^t} = (L^{-3+3r-t}.M^{1-r}.K^{-q}.T^{-s}) = 1$$

Thus, we equate the exponents. So, for example, for the first Pi group, this process results in equations such as:

1+3b-d=0, -b=0, -a=0, and -c=0.

In Matrix formate:

$$\begin{pmatrix} 0 & 3 & 0 & -1 \\ 0 & -1 & 0 & 0 \\ -1 & 0 & 0 & 0 \\ 0 & 0 & -1 & 0 \end{pmatrix} \begin{pmatrix} a \\ b \\ c \\ d \end{pmatrix} = \begin{pmatrix} 0 \\ 0 \\ 0 \\ 1 \end{pmatrix} \quad \text{(S6)}$$

Solving this system gives,

$$\begin{pmatrix} a \\ b \\ c \\ d \end{pmatrix} = \begin{pmatrix} 0 \\ 0 \\ 0 \\ 1 \end{pmatrix}. \quad \text{(S7)}$$

Determining the exponents for all the Pi groups is merely a problem in linear algebra, which has been incorporated into in the supplementary computer code.

Final Dimensionless Equations: The exponents can then substitute into Equation (S4) to obtain expressions for the dimensionless Pi groups and the dependent variable. The resulting equations represent the Buckingham Pi dimension reduction model, where the dependent variable is expressed as a function of the dimensionless Pi groups.

$$U\pi_1 = [P_z/C_{fit}]$$

$$U\pi_2 = [B_t/A]$$

$$U\pi_3 = [P/C_{fit}] \quad \text{(S8)}$$

$$U\pi_4 = [t_o/\mathrm{T}]$$

$$Y_\pi = [EC_{org}/IC_{org}]$$

In our case the dimensionless Pi groups emerge as simple ratios of the original variables.

Now, y_π becomes a function, g say, of $U\pi_1, U\pi_2$, and $U\pi_3$ expressed as

$$Y_\pi = g(U\pi_1, U\pi_2, U\pi_3, U\pi_4) \quad \text{(S9)}$$

The function g is our predictive model. The, N, observations in the training dataset

$[U\pi_1(i), U\pi_2(i), U\pi_3(i), U\pi_4(i), Y_\pi(i)]_{i=1}^{N}$

can then be used to calibrate a function or train an algorithm to derive an appropriate function, g.

Determine the model (function, g): Having reduced the dimensions it remains to determine the function g. For this we can use a wide range of approaches. We can impose a particular functional form, which is the approach traditionally used in physical engineering applications of Buckingham Pi theory; typically, a monomial function is the first to be applied. Alternatively, we can use entirely data driven models, where the ultimate model structure is more or less apparent depending on the methods uses.

For the monomial approach, we assume that the dependent variable Yπ can be expressed as:

$$Y_\pi = \propto_0 \; {U\pi_1}^{\propto_1} {U\pi_2}^{\propto_2} {U\pi_3}^{\propto_3} {U\pi_4}^{\propto_4} \;, \quad \text{(S10)}$$

where, $\{\propto_1, \dots, \propto_4\}$ are arbitrary constants. To estimate these constants based on the training data then we note,

$$\ln(Y_\pi) = \ln(\propto_0) + \sum_{i=1}^{4}\left(\propto_i \ln(U\pi_i)\right) \;, \quad \text{(S11)}$$

This transformation linearizes the relationship between $In(Y_\pi)$, and $In(U_{\pi i})$, enabling us to use linear regression to estimate the values of $\{ \propto_1, \propto_2, \propto_3, \propto_4 \}$. Each $\propto_1$is treated as a coefficient to be learned from the data. To mitigate overfitting and prevent the model from excessively fitting the training data, ridg e regression was applied. Ridge regression regularizes the model by applying a penalty to the size of the coefficients, shrinking them and improving generalizability. To determine the optimal regularization strength, $\propto_1$, a grid search [5] with cross-validation was performed over a range of values for the penalty parameter. The best $\propto_1$ was selected based on the performance of the model in terms of minimizing the mean squared

error during the cross-validation process. By applying ridge regression with the optimal value of $\propto_1$,the model's coefficients were regularized, leading to more stable and robust estimates for the constants$\{ \propto_1, \propto_2, \propto_3, \propto_4 \}$.We use BP-LR to refer to this predictive model. The purely data-driven method that we have adopted is to use a neural network. Using the same reduced non-dimensional training set, we first scale the non-dimensional variables to have a mean of zero and a standard deviation of one. The scaled independent variable vectors then serve as inputs for a three-layer feedforward neural network [6]. This network comprises one input layer, two hidden layers, and one output layer, with ReLU activation functions used for all layers except the output layer, which employs a linear activation function. The architecture of the neural network is defined by the parameters listed in supplementary Table S3 (available in the Supplementary Methods), where the number of seeds is set to 7. This "number of seeds" controls randomization aspects like weight initialization and data shuffling, ensuring consistent results across training runs for reproducibility. Additionally, the Adaptive Moment Estimation (Adam) optimizer is used for training the model. We use a 5-fold cross-validation approach to evaluate the model's performance. In this process, the training dataset is divided into five subsets, and each subset is used once as a validation set while the remaining four subsets are used for training. This fitting method combines training with validation each time the training dataset is presented to the network (epoch), ensuring reliable performance evaluation and preventing overfitting. Additionally, an L2 kernel regularizes is applied to the network to mitigate overfitting and enhance generalization performance. We refer to this application of the neural networks to Environmental Buckingham Pi variables as BP-NN.

Dimensionality Reduction Techniques

Dimensional analysis using the Buckingham Pi theorem was employed to transform physical variables into dimensionless groups, thereby enabling model generalization across varying biofilter scales and configurations. While the Pi theorem ensures physical consistency and scale-independence, additional dimensionality reduction techniques were explored to further enhance model performance and address potential multicollinearity among input features.

In this section, we describe the application of three widely used dimensionality reduction techniques: Principal Component Analysis (PCA), Kernel Principal Component Analysis (KPCA), and Autoencoders. These methods were applied to evaluate their effectiveness in compressing the high-dimensional input space into a lower-dimensional latent representation while retaining key predictive information. The resulting reduced feature sets were

subsequently used to train machine learning models, and their predictive performance was compared against models trained on the full set of dimensionless input variables.

PCA, Kernel PCA, and Autoencoder Models

In this study, we employed three dimensionality reduction techniques: Principal Component Analysis (PCA), Kernel Principal Component Analysis (Kernel PCA), and Autoencoders for feature extraction and data compression. PCA is a classical linear method that transforms data into orthogonal components capturing the maximum variance in the dataset. Kernel PCA extends PCA by using kernel functions to capture nonlinear relationships in the data, allowing for more flexible feature extraction. In contrast, the Autoencoder is a nonlinear neural network-based approach that learns efficient data representations by encoding inputs into a compressed latent space and reconstructing them through decoding. These methods were evaluated for their ability to reduce input dimensionality while preserving essential information for predictive modelling.

PCA for Dimensionality Reduction

Principal Component Analysis (PCA) [7,8] was employed as an unsupervised linear transformation technique to reduce the dimensionality of the feature space while retaining the maximum variance present in the original dataset. PCA projects the original, potentially correlated features X (an m*n data matrix with m samples and n features) onto a new set of orthogonal axes called principal components (PCs), ordered by the amount of variance they explain. Mathematically, PCA solves the eigenvalue decomposition problem of the covariance matrix C:

$$C = \frac{1}{m-1} X^T X$$

where C is an $n \times n$ covariance matrix of the mean-centered data X. The principal components correspond to the eigenvectors v_i of C associated with eigenvalues λ_i, which satisfy:

$$Cv_i = \lambda_i v_i$$

The eigenvalues λ_i represent the variance explained by each principal component i, with the components ordered so that:

$$\lambda_1 \geq \lambda_2 \geq \cdots \lambda_n$$

The data is then projected onto the first N principal components to obtain the reduced dataset:

$$Z = XW$$

where $W = [v_1, v_2, \dots, v_N]$ is the matrix of selected eigenvectors.

In this study, the number of retained components was set to $N = 4$ to maintain consistency with EnviroPiNet, which also reduces the feature space to four variables for dimensionality reduction. The input dataset consisted of a training set, where the features were normalized using min-max scaling:

$$x' = \frac{x - x_{min}}{x_{max} - x_{min}}$$

to ensure that each variable contributed equally to the analysis. PCA was applied to the normalized feature space using the pca function from the data analysis module, and the first four principal components were retained.

To evaluate the PCA transformation, we examined the loadings of each original feature on the principal components and quantified the variance explained by each component. The loadings indicate the correlation of each original feature with the PCs, helping interpret the influence of variables. For example, PC1 showed strong positive loadings from *Cfit* (0.4521) and *Pv* (0.4521), and a strong negative loading from *P* (−0.4521), suggesting opposing influence patterns. These results are presented in **Table S3**. The percentage of variance captured by each PC was computed as:

$$Variance\ explained\ by\ PC_i = \frac{\lambda_i}{\sum_{j=1}^{n} \lambda_j} \times 100\%$$

And was: *PC1*(67.62%), *PC2* (14.35%), *PC3*(7.98%), and *PC4*(7.37%), collectively accounting for 97.32% of the total variance. These variance contributions are illustrated in Fig.S2. Subsequently, a feedforward neural network was trained on the PCA-compressed features. The architecture and hyperparameters of the neural network were consistent with those described in Table S4. The model was trained on the transformed training dataset and evaluated on an independent test set. Model performance was assessed using the coefficient of determination (R^2 score) and symmetric Mean Absolute Percentage Error (sMAPE). The training R^2 was 0.90, and the test R^2 was 0.54. Additionally, a regression model was applied to

the PCA-reduced features for comparative analysis. The results are summarized in Tables 1 and 2**.**

Table S3. PCA loadings matrix showing the correlation between the original features and the four retained principal components (PC1–PC4). These loadings indicate how strongly each original variable contributes to each principal component.

Variables	*PC1*	*PC2*	*PC3*	*PC4*
T	-0.2105552	-0.7864633	-0.2259461	-0.5287462
IC_{org}	-0.3389534	0.23635182	-0.6956617	0.18269891
A	0.37030973	-0.2799637	-0.4388071	0.27779067
B_t	-0.3007377	-0.4715052	0.3016072	0.77414595
P	-0.4521079	0.09113448	0.02224531	-0.0593829
C_{fit}	0.45210789	-0.0911345	-0.0222453	0.05938292
t_o	0	0	0	0
P_z	0.45210789	-0.0911345	-0.0222453	0.05938292

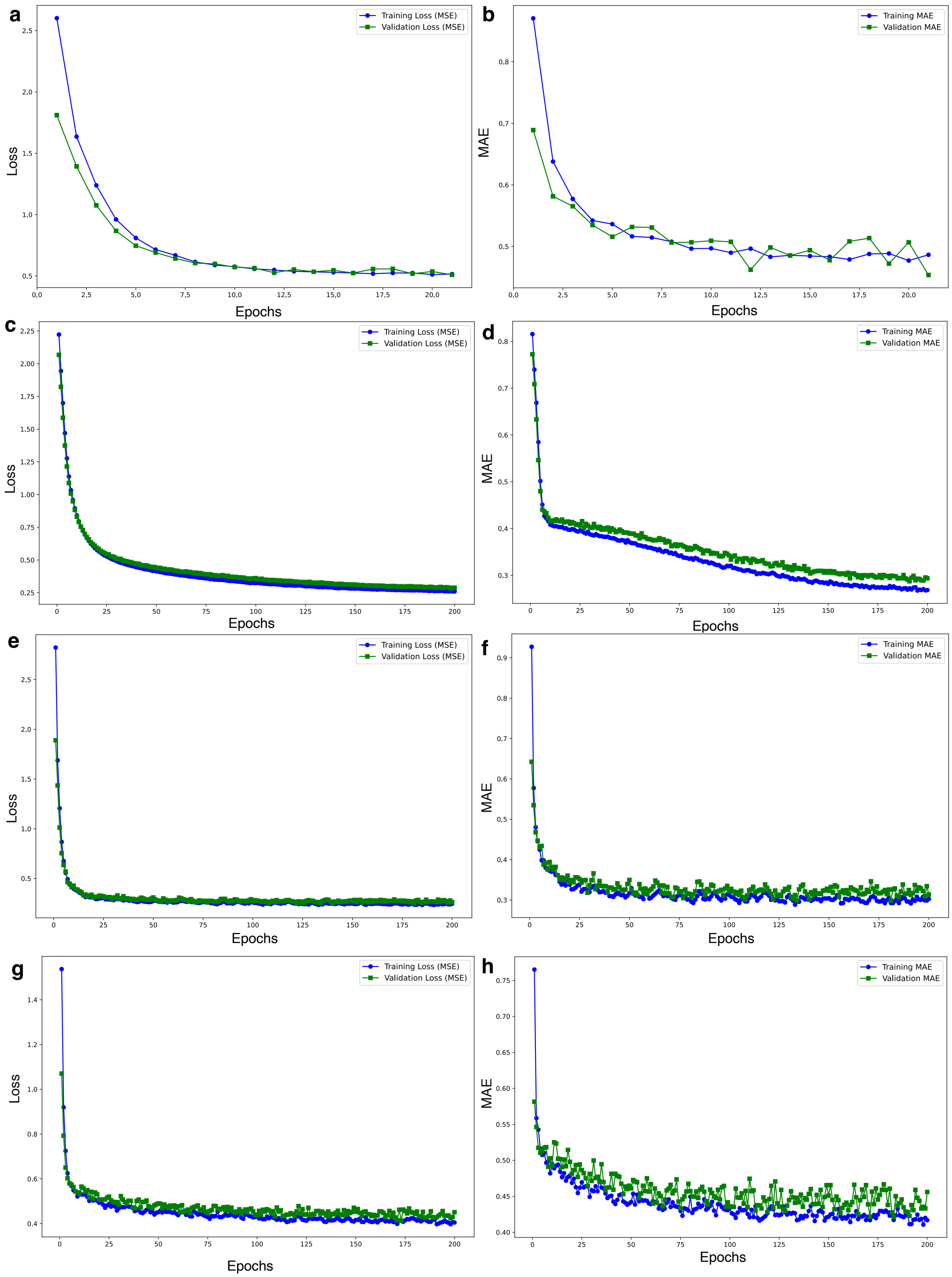

Fig.S1: Learning curves and performance metrics (MSE and MAE) for EnviroPiNet, Autoencoder-NN, PCA-NN, and KPCA-NN, illustrating learning behavior and predictive

performance for Patient 10. **a**, Mean Squared Error (MSE) learning curve for EnviroPiNet, showing both training and validation losses decreasing and converging, with early stopping at epoch 21 indicating effective learning and strong generalization. **b**, Mean Absolute Error (MAE) for EnviroPiNet, reflecting improved predictive accuracy. **c, e, g**, MSE learning curves for Autoencoder-NN, PCA-NN, and KPCA-NN. The Autoencoder-NN demonstrates a consistent decrease in both training and validation losses, suggesting ongoing learning but with a risk of overfitting. PCA-NN's, and KPCA-NN losses drop rapidly and then plateau, reflecting its linear nature and limited capacity for further learning. **d, f, h**, MAE performance for Autoencoder-NN, PCA-NN, and KPCA-NN. While Autoencoder-NN achieves lower MAE, its steady loss reduction may indicate overfitting. KPCA-NN exhibits a similar convergence pattern to PCA-NN, with losses dropping quickly and then plateauing, reflecting comparable generalization limitations despite KPCA's capacity to capture some nonlinearity.

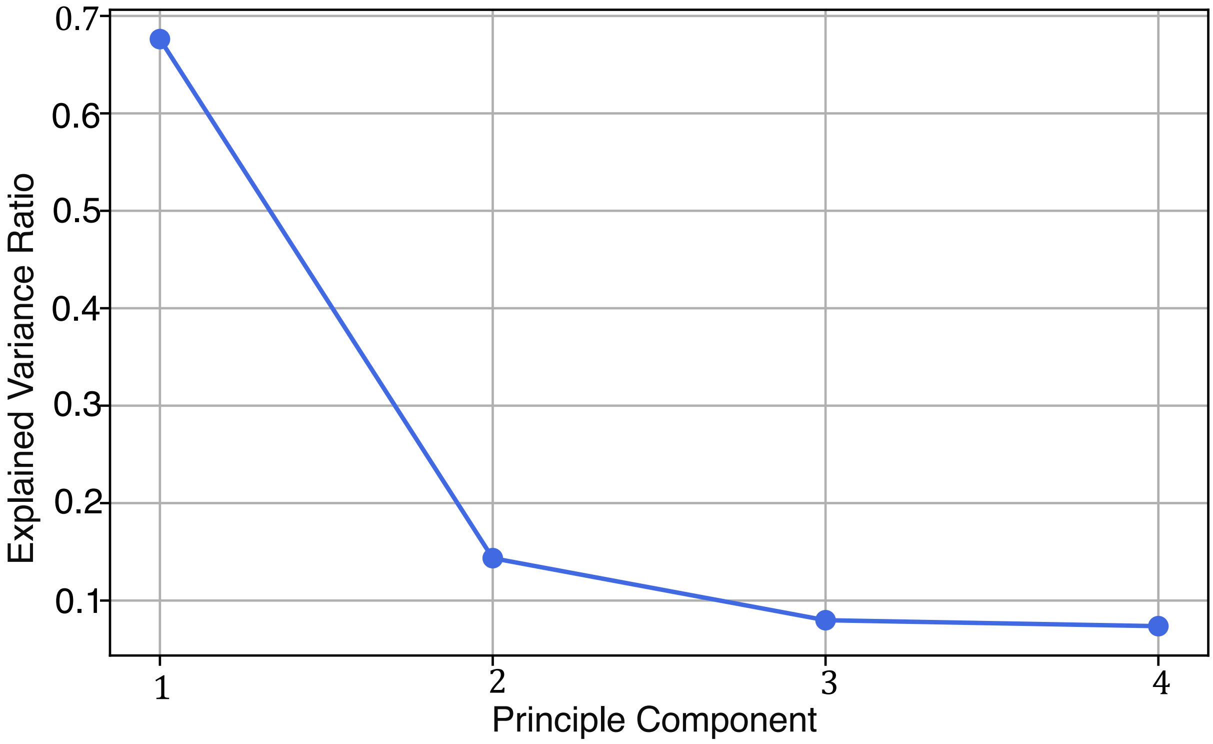

Fig.S2. Scree plot showing the percentage of variance explained by each of the first four principal components. PC1 explains the majority of the variance (67.62%), followed by PC2 (14.35%), PC3 (7.98%), and PC4 (7.37%), collectively accounting for 97.32% of the total variance. This plot illustrates the effectiveness of dimensionality reduction using PCA.

Autoencoder Architecture and Training

The Autoencoder [9,10] was implemented as a feedforward neural network consisting of an input layer, multiple hidden layers, a bottleneck (latent) layer, and a symmetric decoder.

The encoder compresses the input data into a lower-dimensional representation at the bottleneck layer, while the decoder reconstructs the original input from this compressed encoding. The network architecture included 8 input neurons, followed by hidden layers with 64, 32, 16, and 8 neurons, and a latent space (bottleneck) dimension of 4. The decoder mirrored the encoder structure in reverse. ReLU activation functions were used for the hidden layers, a tanh function for the bottleneck layer, and a linear activation for the output layer. The model was compiled using the Adam optimizer with a learning rate of 0.001 and trained to minimize the mean squared error (MSE) loss between the input and its reconstruction. The overall structure is illustrated in Fig.S3.

The compressed features obtained from the Autoencoder were subsequently used as input for a feedforward neural network. The hyperparameters used for this neural network are listed in Table S4. Except for the learning rate and dropout rate which were set to **0.001** and **0.2**, respectively all other parameters were kept the same as specified in the table. The neural network was trained on the training dataset and evaluated on an independent test dataset. Its performance was assessed using the coefficient of determination (R^2 score) and the symmetric Mean Absolute Percentage Error (sMAPE) as performance metrics. In addition, a regression model was also applied to the compressed features to compare performance. The results are summarized in Table 1 and 2.

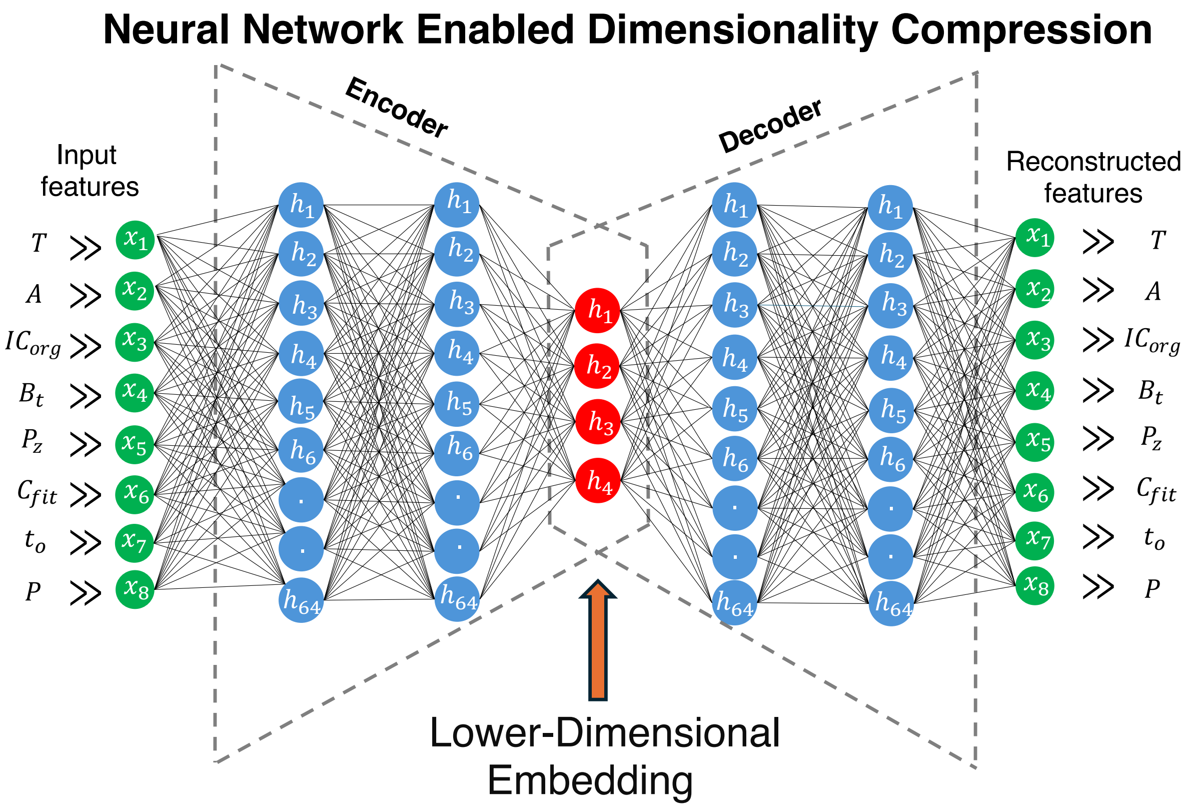

Fig.S3: Autoencoder-Based Dimensionality Reduction. The Autoencoder model reduces the input data from 8 variables to 4 latent variables. As described in the main manuscript, these input variables include T (temperature), A (filter age), IC_{org} (influent carbon concentration), B_t (empty bed contact time), P_z (pore size), P (particle size), C_{fit} (filter diameter), and t_o (ambient temperature). The effluent carbon concentration (EC_{org}) is modeled as the output. The Autoencoder compresses the inputs into a latent space while ensuring that the original data can be reconstructed. These latent variables can then be used to model biofilter performance or similar systems.

Table S4: Hyperparameters used for model training.

**Parameter**	**Value**
Batch size for training	16
Learning rate for optimization	0.05
Number of units in each layer	64
Total number of layers (1 input, 2 hidden)	3
Number of training epochs	200

Number of splits for cross-validation	5
Activation function	ReLU
L2 regularization parameter	0.01
Number of random seeds for reproducibility	7
Dropout rate	0.1

Note: The above hyperparameters were selected through a combination of manual tuning and randomized grid search, using performance on 5-fold cross-validation as the primary selection criterion. Values were chosen within reasonable bounds informed by prior experience and relevant literature. Early stopping with a patience of 10 epochs was employed to prevent overfitting.

Kernel PCA for Nonlinear Dimensionality Reduction

Kernel PCA [11] was utilized to perform nonlinear dimensionality reduction, enabling the extraction of informative low-dimensional representations from complex input data. Unlike classical PCA, which identifies linear projections that maximize variance, Kernel PCA leverages kernel functions to implicitly map input features into a higher-dimensional space, thereby capturing nonlinear relationships. In this study, we applied Kernel PCA using the Radial Basis Function (RBF) kernel [12], which is particularly effective in modelling nonlinear data structures. The number of components was set to four, aligning with the reduced dimensionality used in PCA and Autoencoder-based approaches to facilitate fair comparisons. The kernel parameter γ was left at its default setting, which corresponds to the reciprocal of the number of input features. Additionally, the fit_inverse_transform=True option was enabled to allow reconstruction of the original feature space from the reduced dimensions, improving interpretability. Before applying Kernel PCA, all features were standardized using StandardScaler, which transforms each feature to have zero mean and unit variance. This standardization ensures that all variables contribute equally to the kernel computation and prevents dominance by features with larger scales.

The reduced feature representations obtained from Kernel PCA were subsequently used to train both a feedforward neural network and a set of regression models to assess predictive performance. The neural network architecture and training hyperparameters were consistent with those described in Table S4, ensuring comparability across dimensionality reduction

techniques. Model performance was evaluated on the training and independent test datasets using the **c**oefficient of determination (R^2) and symmetric Mean Absolute Percentage Error (sMAPE) as evaluation metrics. The results of both modelling approaches are presented in Tables 1 and 2, enabling direct comparison with PCA and Autoencoder-based methods.

SHAP Analysis Setup

SHapley Additive exPlanations (SHAP) [13] were used to interpret the contribution of individual features to the Gradient Boosting Regressor model's predictions. We employed the shap.Explainer with the trained GradientBoostingRegressor (gb_model) and the input dataset. The explainer was applied to the training dataset to compute SHAP values representing each feature's additive impact on the predictions. Waterfall plots visualize how each feature contributes to the model's prediction for a given observation. Here, $E[f(x)]$ denotes the average predicted effluent organic carbon concentration (EC_{org}).) across all observations, while $f(x)$ is the prediction for a specific observation. The SHAP values explain how each feature shifts the prediction away from the average. For example, a particular Buckingham Pi group feature may increase the predicted concentration by a certain amount, indicating its influence for that observation. Summary plots display the distribution of SHAP values for all observations across each feature, showing both the magnitude and direction of each feature's impact on the model output. These plots help identify which features have the greatest influence and whether their effects tend to increase or decrease the predicted value. Bar plots show the mean absolute SHAP values for each feature, providing a straightforward ranking of overall feature importance based on average contribution magnitude, regardless of direction. Together, these plots allowed us to identify which Buckingham Pi groups had the strongest positive or negative influence on the predicted effluent organic carbon concentration (EC_{org}).

Ablation Study Configuration

To evaluate the contribution of physics-based Buckingham Pi groups, an ablation study was conducted by training the same neural network architecture using the original raw input variables without applying the Buckingham Pi transformation. The neural network was trained on the identical training set and optimized using the same hyperparameters as the EnviroPiNet model. Model performance was assessed on the test set using metrics such as the coefficient of determination (R^2), and symmetric Mean Absolute Percentage Error (sMAPE). Comparing these metrics between the model trained on raw features and the model trained on Buckingham

Pi features allowed us to isolate the effect of incorporating physics-guided feature engineering on predictive accuracy. The results, presented in the Supplementary Results section, show a clear improvement in predictive skill when Buckingham Pi groups are included, demonstrating the added value of physics guidance in the modelling framework.

Supplementary Results

Performance Difference Between Autoencoder-NN and Autoencoder-LR

To understand the substantial difference in performance between Autoencoder-NN and Autoencoder-LR models (Table 1), we analyzed their average R^2 values over 10 runs. The Autoencoder-NN model consistently achieved a moderate R^2 of 0.2, while Autoencoder-LR performed poorly with an average R^2 of –0.21. This discrepancy is due to the nonlinear nature of the latent features generated by the autoencoder. Linear regression is limited to modelling linear relationships and fails to capture the complex, nonlinear structures present in the latent space. In contrast, the neural network can learn and utilize these nonlinear patterns effectively, resulting in better predictive accuracy. This highlights the importance of aligning model complexity with the characteristics of the input feature space.

Table S5: Performance metrics for the EnviroPiNet neural network trained on Buckingham Pi transformed inputs versus raw input variables. Metrics include coefficient of determination (R^2) for training and testing sets, and symmetric Mean Absolute Percentage Error (sMAPE). The model using Buckingham Pi features shows improved predictive skill compared to the model trained on raw variables.

Model	Input Type	Train R^2	Test R^2	Train sMAPE	Test sMAPE
EnviroPiNet	Buckingham Pi	0.94	0.9	2.1	5.6
EnviroPiNet	Raw variables	0.893	-0.977	4.24	19.332

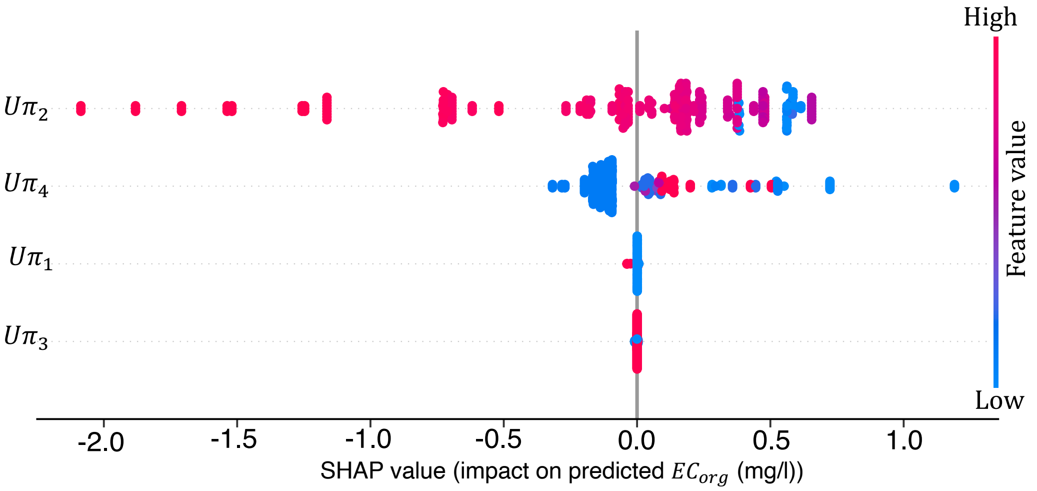

Fig.S4: SHAP summary plot of feature importance for the Gradient Boosting Regressor models trained on Buckingham Pi groups using the training dataset. Each point represents a SHAP value for a feature and sample, showing the distribution of feature impacts on predicted carbon concentration EC_{org}.

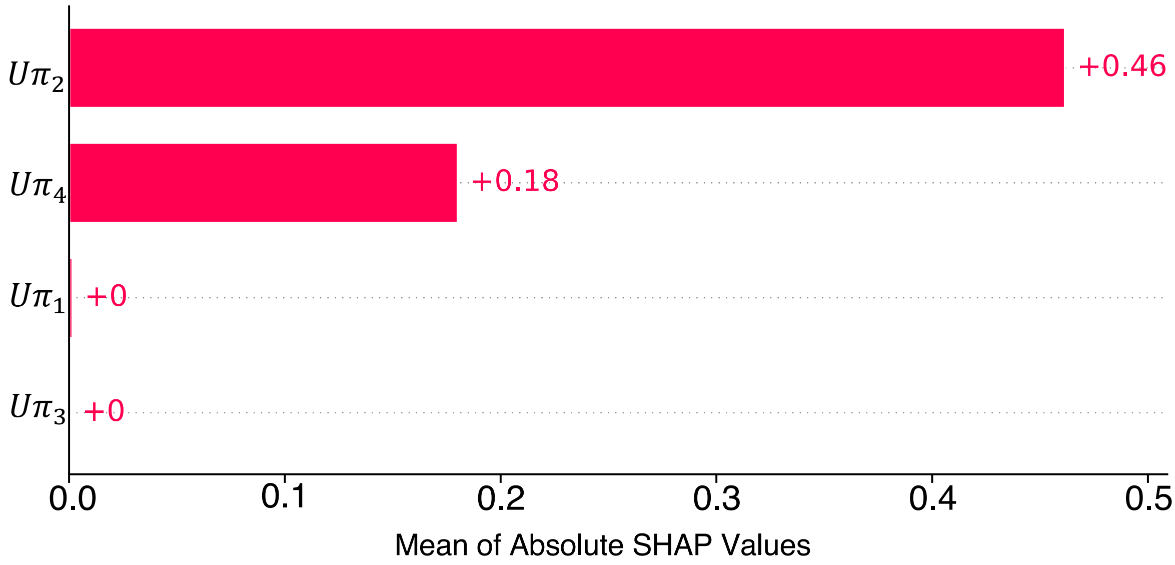

Fig.S5: Bar plot of mean absolute SHAP values for Buckingham Pi features, illustrating the overall importance of each feature in the Gradient Boosting Regressor model's predictions on the training data. Features $U\pi_2$ and $U\pi_4$ exhibit the highest average contributions.

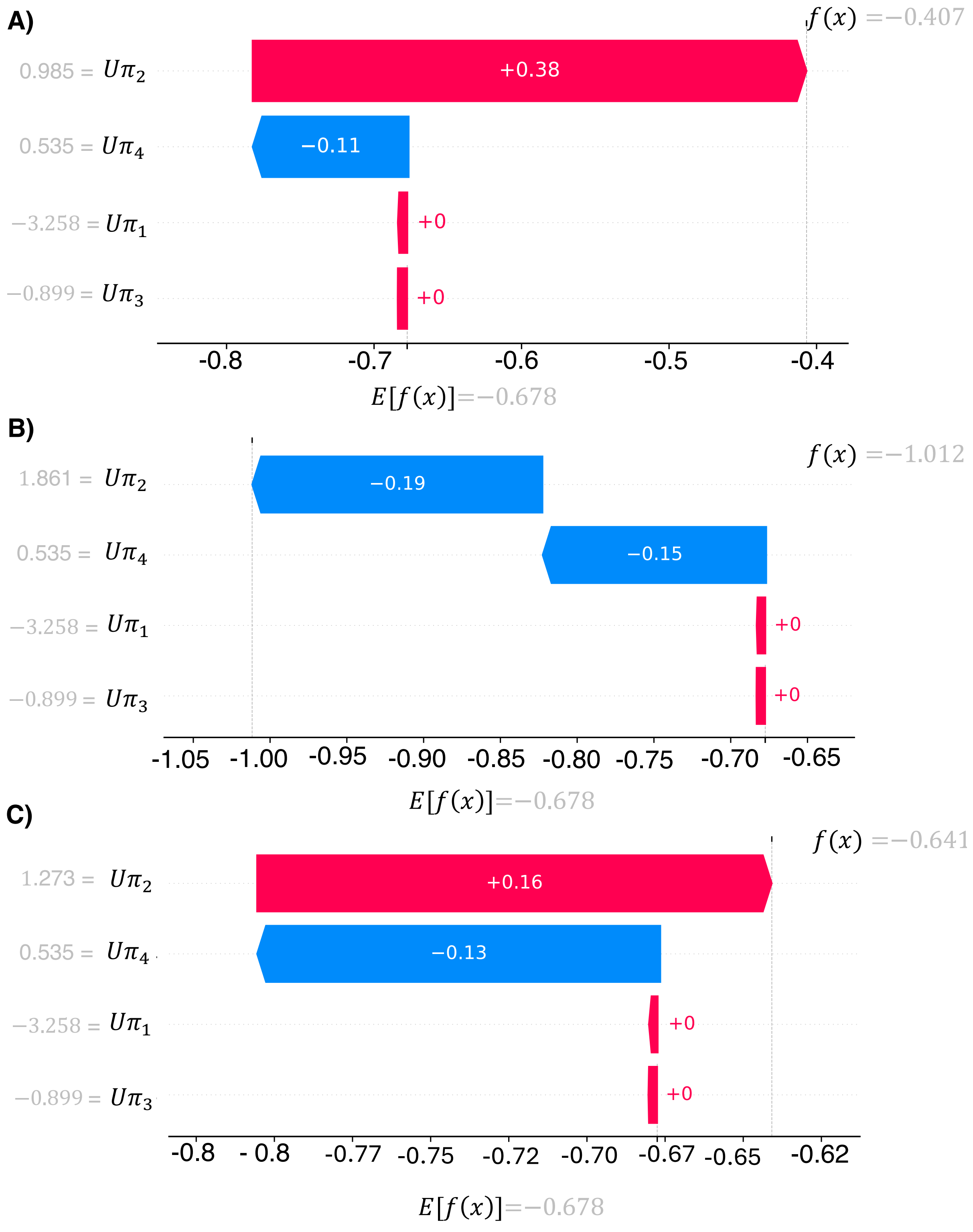

Fig.S6. Waterfall plots depicting SHAP values for individual observations from the training dataset: **(A)** sample 0, **(B)** sample 10, and **(C)** sample 25. The x-axis shows the magnitude of each feature's contribution to the prediction, with red bars indicating positive contributions (increasing the prediction) and blue bars indicating negative contributions (decreasing the prediction). The y-axis lists the Buckingham Pi features. These

plots illustrate how the model's predictive value $f(x)$ for each sample is built by adding individual SHAP contributions to the base value $E[f(x)]$. Notably, in A) and B), $U\pi_2$ and $U\pi_4$ show both positive and negative contributions, while in (B), both features contribute negatively. This highlights variation in feature influence across samples.

References:

[1] Quinn, A. *Investigating the Effects of Biofilter Design on Bacterial Communities in Drinking Water Treatment*. [PhD thesis, University of Glasgow]. Glasgow, United Kingdom, (2024).

[2] Ma, B., LaPara, T.M. and Hozalski, R.M. Microbiome of drinking water biofilters is influenced by environmental factors and engineering decisions but has little influence on the microbiome of the filtrate. *Environmental science & technology*, *54*, 1526-11535 (2020).

[3] Shi, X., Pereira, R., Savage, L., Poursat, B., Quinn, D., Kostrytsia, A., Cholet, F., Smith, C.J., Gauchotte-Lindsay, C., Sloan, W.T. and Ijaz, U.Z. Microbial stratification and DOM removal in drinking water biofilters: Implications for enhanced performance. *Water Research*, *262*, 122053 (2024).

[4] Oppenheimer, M.W., Doman, D.B. and Merrick, J.D., 2023. Multi-scale physics-informed machine learning using the Buckingham Pi theorem. *Journal of Computational Physics*, *474*, p.111810.

[5] Krstajic, D., Buturovic, L.J., Leahy, D.E. and Thomas, S., 2014. Cross-validation pitfalls when selecting and assessing regression and classification models. *Journal of cheminformatics*, *6*(1), p.10.

[6] Chicco, D., Warrens, M.J. and Jurman, G., 2021. The coefficient of determination R-squared is more informative than SMAPE, MAE, MAPE, MSE and RMSE in regression analysis evaluation. *Peerj computer science*, *7*, p.e623.

[7] Uzma & Halim, Z. An ensemble filter-based heuristic approach for cancerous gene expression classification. Knowledge-Based Syst. 234, 107560 (2021).

[8] Jolliffe, I. T. Choosing a subset of principal components or variables. Princ. component analysis 111–149 (2002).

[9] Uzma, A.-O. F., Tubaishat, A., Shah, B. & Halim, Z. Gene encoder: A feature selection technique through unsuperviseddeep learning-based clustering for large gene expression data. Neural Comput. Appl. 1, 1–23 (2020).

[10] Uzma, M. U. & Halim, Z. Protein encoder: An autoencoder-based ensemble feature selection scheme to predict protein secondary structure. Expert. Syst. with Appl. 213, 119081 (2023).

[11] Schölkopf, B., Smola, A. & Müller, K.-R. Kernel principal component analysis. In International conference on artificial neural networks, 583–588 (Springer, 1997).

[12] Tao, K.M., 1993, November. A closer look at the radial basis function (RBF) networks. In *Proceedings of 27th Asilomar conference on signals, systems and computers* (pp. 401-405). IEEE.

[13] Lundberg, S.M. and Lee, S.I., 2017. A unified approach to interpreting model predictions. *Advances in neural information processing systems*, *30*.
